# 908. Clinical Characteristics and Outcomes of Cytomegalovirus Disease in HIV-Negative Non-Transplant Patients, a Propensity Score Match Analysis

**DOI:** 10.1093/ofid/ofad500.953

**Published:** 2023-11-27

**Authors:** Ixchel T Salter, Michaele-Francesco Corbisiero, Daniel B Chastain, Jose G Montoya, Raymund R Razonable, Andrés F Henao Martínez

**Affiliations:** University of Colorado, Aurora, Colorado; University of Colorado, Aurora, Colorado; University of Georgia College of Pharmacy, Albany, Georgia; Dr. Jack S. Remington Laboratory for Specialty Diagnostics, Palo Alto Medical Foundation, Sutter Health, Palo Alto, CA, USA, Palo Alto, California; Mayo Clinic, Rochester, Minnesota; University of Colorado Anschutz Medical Campus, Aurora, CO

## Abstract

**Background:**

Cytomegalovirus (CMV) infects millions in the US and is a source of comorbidity among immunocompromised patients. Despite its prevalence, there is limited data on clinical characteristics or outcomes among HIV-negative, non-transplant (NHNT) patients in population-based cohorts.

We aim to evaluate a multicenter US-based network to obtain clinical characteristics and outcomes of NHNT patients with CMV disease and explore potential prognostic clinical characteristics implicated in increased morbidity and mortality.

**Methods:**

This is a US-based multicenter, population-based, retrospective cohort study. We queried TrinetX, a global research network, to identify patients with a CMV disease by ICD-10-CM codes and serum and PCR > 5000 IU/mL. We captured comorbidities diagnosed within 90 days of CMV infection diagnosis, symptoms noted within 30 days, and outcomes of interest at 30 days and 1 year. Primary outcome was all-cause mortality. Secondary outcomes were hospitalization and CMV disease-related complications. We performed a propensity score-matched analysis comparing clinical characteristics among patients who survived versus non-survivors at 90 days.

**Results:**

We found 1123 patients with evidence of CMV disease. The mean patient age was 53 years, 48% men and 50% white. Aplastic anemia and neoplasms were the most common comorbidities (Table 1). Thromboembolism was a common complication (15%). Half of the patients had dyspnea, fever, abdominal pain, and nausea. CMV disease was associated with 14%, 22%, and 26% mortality at 30 days, 90 days, and one year, respectively. 35% of patients required hospitalization within 30 days. After propensity score matching, we had 278 patients in each cohort. Dyspnea and weakness were linked to increased 90-day mortality (Figure 1). Purpura, encephalopathy, and sepsis were more common in patients who died. High CMV serum viral load and ferritin were associated with increased mortality.

**Figure:**
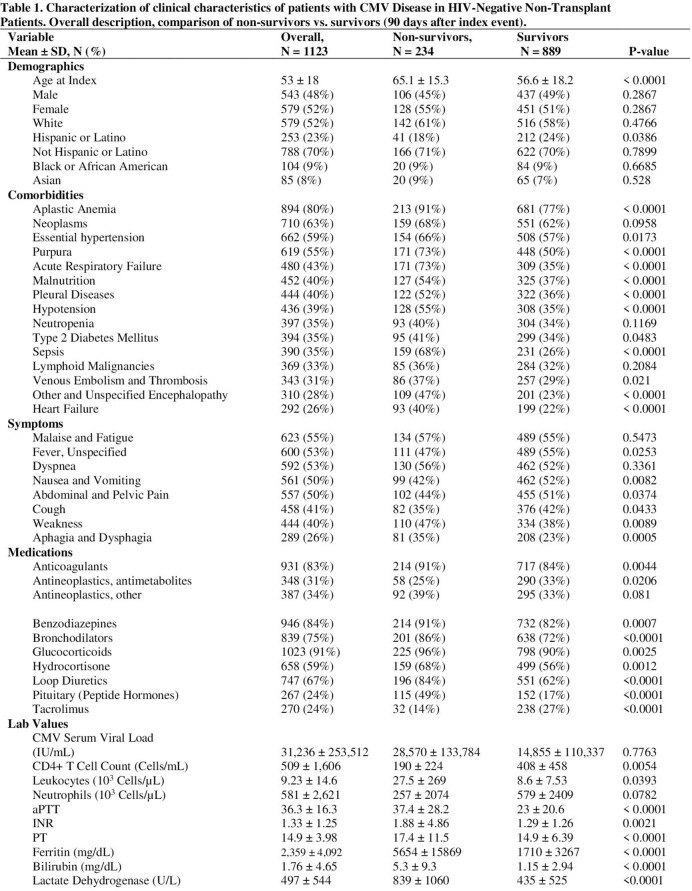

**Figure:**
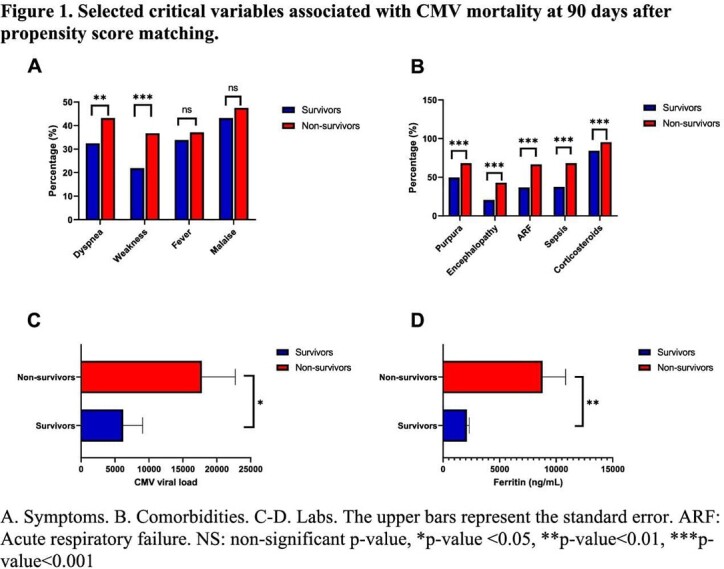

**Conclusion:**

NHNT patients with CMV disease often have underlying comorbidity, commonly aplastic anemia, and neoplasms. CMV disease was associated with thrombosis and a high risk of hospitalization and mortality. Vascular disease, sepsis, high viral load, and cell lysis markers were prognostic factors for mortality in this cohort.

**Disclosures:**

**Raymund R. Razonable, MD**, Allovir: Endpoint Adjudication Committee|American Society of Transplantation: Board Member|Gilead: Grant/Research Support|Novartis: DSMB|Regeneron: Grant/Research Support|Roche: Grant/Research Support

